# Lidocaine, Dexmedetomidine and Their Combination Reduce Isoflurane Minimum Alveolar Concentration in Dogs

**DOI:** 10.1371/journal.pone.0106620

**Published:** 2014-09-18

**Authors:** Carlos M. Acevedo-Arcique, José A. Ibancovichi, Julio R. Chavez, Eduardo Gutierrez-Blanco, Rafael Moran-Muñoz, José M. Victoria-Mora, Francisco Tendillo-Cortijo, Martín Santos-González, Pedro Sanchez-Aparicio

**Affiliations:** 1 Department of Anaesthesia and Analgesia, Faculty of Veterinary Medicine, Universidad Autónoma del Estado de México, Toluca, State of Mexico, Mexico; 2 Department of Orthopaedic Surgery and Trauma, Faculty of Veterinary Medicine, Universidad Autónoma del Estado de México, Toluca, State of Mexico, Mexico; 3 Department of Animal Health and Preventive Medicine, Faculty of Veterinary Medicine, Universidad Autónoma de Yucatán, Merida, State of Yucatan, Mexico; 4 Hospital Universitario Clínica Puerta de Hierro, Madrid, España; Colorado State University, United States of America

## Abstract

The effects of intravenous (IV) lidocaine, dexmedetomidine and their combination delivered as a bolus followed by a constant rate infusion (CRI) on the minimum alveolar concentration of isoflurane (MAC_ISO_) in dogs were evaluated. Seven healthy adult dogs were included. Anaesthesia was induced with propofol and maintained with isoflurane. For each dog, baseline MAC (MAC_ISO/BASAL_) was determined after a 90-minute equilibration period. Thereafter, each dog received one of the following treatments (loading dose, CRI): lidocaine 2 mg kg^−1^, 100 µg kg^−1^ minute^−1^; dexmedetomidine 2 µg kg^−1^, 2 µg kg^−1^ hour^−1^; or their combination. MAC was then determined again after 45- minutes of treatment by CRI. At the doses administered, lidocaine, dexmedetomidine and their combination significantly reduced MAC_ISO_ by 27.3% (range: 12.5–39.2%), 43.4% (33.3–53.3%) and 60.9% (46.1–78.1%), respectively, when compared to MAC_ISO_/_BASAL_. The combination resulted in a greater MAC_ISO_ reduction than the two drugs alone. Their use, at the doses studied, provides a clinically important reduction in the concentration of ISO during anaesthesia in dogs.

## Introduction

The end-tidal partial pressure of inhalant anaesthetics required to prevent movement in 50% of individuals exposed to a supramaximal noxious stimulus (i.e. minimum alveolar concentration, MAC) represents an index of potency of anaesthetic agents [1]. The MAC of contemporary inhalant anaesthetics has been previously reported in dogs and after the administration of different opioids, sedatives, tranquilisers and local anaesthetics [2], [3], [4]. Clinically, one of the main issues concerning inhalant anaesthesia is the progressive cardiovascular depression related to the delivery of high concentrations. Drugs such as lidocaine (local anaesthetic) and dexmedetomidine (sedative) decrease the MAC of inhaled anaesthetics and may also reduce the risk of cardiopulmonary depression by means of decreasing the inhalant anaesthetic requirements during anaesthesia [5], [6], [7]. In addition, the combination of these agents with different pharmacological mechanisms of action may provide better analgesia and an even a greater inhalant-sparing effect [2], [8], [9], [10], [11]. Lidocaine (LIDO) is an amide local anaesthetic that can be administered intravenously (IV) via a bolus or constant rate infusion (CRI) to provide perioperative analgesia, sedation and anti-arrhythmic effects. In dogs, lidocaine decreases the MAC of inhaled anaesthetics by 18.7% to 43.3% in a dose-dependent manner [2], [3], [5], [7], [9], [12], [13]. Dexmedetomidine (DEX) is the active enantiomer of the racemic mixture medetomidine. DEX is a potent α_2_ adrenoceptor agonist; it is approximately eight times more selective toward α_2_ adrenoceptors than clonidine [14] and at least two times as potent as medetomidine [15]. It has a high α_2_: α_1_ selectivity ratio (1620∶1) compared with xylazine (160∶1), clonidine (220∶1), and romifidine (340∶1) [16]. DEX is widely used in small animal anaesthesia to provide sedation, anxiolysis and analgesia [6]. DEX bolus, CRI and epidural administration has been shown to reduce the anaesthetic requirement for the induction and maintenance of general anaesthesia in dogs [4], [6], [17]. To the best of our knowledge, the effect of a LIDO-DEX combination administered by bolus followed by CRI on the MAC_ISO_ has not been reported in dogs. The purpose of this study was to evaluate the MAC_ISO_-sparing effect of LIDO, DEX and their combination in dogs. Bearing in mind their different mechanisms of action, the authors hypothesised that the combination LIDO-DEX would reduce MAC_ISO_ to a greater extent than the two drugs alone.

## Materials and Methods

This study was approved by the Animal Research Ethics Committee for Animal Experimentation (protocol number 2267/2009) of the Faculty of Veterinary Medicine, Universidad Autónoma del Estado de México.

### Animals

Seven adult (1 to 2 years old) mixed-breed neutered dogs (three male and four female; mean ± SD body weight of 18.1±9 kg) were included in a prospective randomised blinded cross-over experiment with a two-week washout period between treatments. A computer generated random numbers table was used to assign treatments. Each dog was studied on three separate occasions, receiving one treatment at one time. The treatments were assigned in random order. Dogs were considered to be healthy based on medical history, physical examination, complete blood count and serum biochemical analysis and urinalysis. Food, but not water, was withheld 8 hours prior to each anaesthetic procedure.

### Anaesthesia and instrumentation

A 20-gauge catheter was aseptically placed into the cephalic vein, and connected to a resealable male luer injection port (BD-luer loK; Becton Dickinson and Company, NJ, USA). Anaesthesia was induced with IV administration of propofol to effect (4–6 mg kg^−1^; Fresofol 1%; Fresenius Kabi, Australia). After intubation was performed with an appropriately sized, cuffed endotracheal tube, dogs were connected to a rebreathing system. Anaesthesia was maintained with isoflurane (ISO) (Forane; Baxter Laboratories, USA) and oxygen flow rates were set at 100 mL kg^−1^ minute^−1^ after induction; 10 minute later, the oxygen flow was reduced to 50 mL kg^−1^ minute^−1^. Intermittent positive pressure ventilation (IPPV) was instituted (Fabius Dräger Medical; Lübeck, Germany), and the respiratory rate and inspiratory peak airway pressure were adjusted to maintain eucapnia (end-tidal carbon dioxide tension [PÉCO_2_] 33–45 mmHg, 4.4–5.9 kPa). Dogs were then placed in lateral recumbency and a 20-gauge catheter (Introcan; B-Braun, Brazil) was aseptically introduced into the dorsal pedal artery for direct blood pressure monitoring and the collection of arterial blood to determine blood gases. A thermistor was advanced to the thoracic portion of the oesophagus for body temperature monitoring. A circulating warm-water blanket was used to maintain body temperature at 37.5–38.5°C. An electrolyte solution (saline 0.9%; Solution DX-CS; Pisa Farmaceutica, Mexico) was administered at 3 mL kg^−1^ hour^−1^ throughout anaesthesia by the use of an infusion pump (Colleague; Baxter Healthcare Corporation Medication Delivery, IL, USA).

Heart rate and rhythm were obtained by a continuous lead II ECG trace (Surgivet; Smith Medical Inc., Waukesha, WI, USA). Systolic, mean and diastolic arterial blood pressures (SAP, MAP and DAP, respectively) were continuously monitored via a blood pressure transducer system (BD DTX Plus; Becton Dickinson and Company) connected to the dorsal pedal artery. Before each experiment, the transducers was zeroed, calibrated and levelled to the heart position. Zeroing was performed by exposing the transducer to atmospheric pressure and levelled to the heart position. The calibration was performed using a mercury manometer as the gold standard.

Pulse oximetry (SpO_2_) was measured by pulse oximetry (Advisor; Surgivet) with a sensor attached to the dog’s tongue. Inspired ISO (FÍIso) and end-tidal (FÉIso) concentrations, PÉCO_2_ and respiratory rate (*f*_R_) values were continuously measured with an infrared gas analyser (Dräger Vamos; Dräger Medical, Lübeck, Germany). The gas analyser was calibrated before starting each experiment with a standard gas mixture provided by the manufacturer. For blood gas analysis (pH, PaO_2_ and PaCO_2_), 0.5 mL of blood were obtained from the catheter placed in the dorsal metatarsal artery immediately before the first noxious stimulation and another one after determining the MAC. The inspired oxygen fraction and temperature were corrected (GEM Premier 3000; Instrumentation Laboratory, UK). The gas analyser was calibrated before each experiment by using two aqueous buffered bicarbonate solutions containing precise concentrations of carbon dioxide and oxygen (GEM CVP Solutions 1–2: Instrumentation Laboratory, UK).

### MAC determination

Following propofol induction, the dogs were anaesthetised for at least 90 min as an initial equilibration period at an ET_ISO_ of 1.8% to minimise the effects of propofol. The determination of MAC_ISO/BASAL_ for each dog was started after the initial equilibration period. Once the MAC_ISO/BASAL_ was determined, the dogs received a bolus followed by CRI treatment of lidocaine, or dexmedetomidine, or their combination. The CRI of drugs (LIDO, DEX and LIDO-DEX) was maintained for 45 min allowing for an adequate, theoretical plasma drug equilibration time based on prior investigations [3], [18]; after this period, the isoflurane treatment MAC (MAC_ISO/T_) was determined and the CRI of drugs was stopped.

Determinations of MAC were carried out using well-established techniques [3]. Noxious stimulation was applied by clamping a paw at the fourth digit. The clamping technique was performed with 24-cm sponge forceps (with protective plastic tubing on each jaw) clamped to the first notch until gross purposeful movement was detected or a period of 60 sec elapsed [19]. A positive motor response was considered if jerking or twisting motion of the head or if movement of extremities was observed. A negative response included a lack of movement of the head and limbs, muscle rigidity, shivering, tail movement, swallowing, chewing or an increase in spontaneous respiratory efforts during controlled ventilation [19], [20]. When a positive response was elicited, the ET_ISO_ was increased by 10% and maintained at this concentration for at least 20 min, and the noxious stimulus procedure was repeated. When a negative response was detected, the ET_ISO_ was decreased by 10% and maintained at this concentration for at least 20 minute, and the noxious stimulus procedure was repeated. The procedure was continued until purposeful movement ceased (after an increase in the anaesthetic concentration) or returned (after a decrease in the anaesthetic concentration). The isoflurane MAC was calculated as the mean value between the highest ET_ISO_ at which the purposeful movement was detected and the lowest ET_ISO_ at which the purposeful movement was not detected. In each dog, the MAC basal and MAC treatment were evaluated in duplicate and averaged.

The isoflurane MAC values were corrected to sea level by multiplying the barometric pressure of the location/760 mmHg by the obtained MAC value. The mean barometric pressure was obtained from the official city meteorological station for the altitude at which the experiment was performed (2,680 meters above sea level) and was 556 mmHg. After determining the MAC of the treatments, the CRI of the drugs was discontinued; the dogs were disconnected from the anaesthesia machine, and extubated when the swallowing reflex was present. After recovery, the dogs were administered 4 mg kg^−1^ of carprofen (Rimadyl, Pfizer Animal Health BV, Capelle a/d I Jssel, The Netherlands) subcutaneously every 24 h for two days.

### Experimental protocol

Each dog was anaesthetised on three separate occasions with a two-week washout interval between treatments. Following MAC_BASAL_ determination, the dogs were assigned to one of the following three treatments in a randomised cross-over study design:

Group LIDO: Lidocaine (Lidocaine 2%; Pisa, Mexico) IV loading dose (LD) of 2 mg kg^−1^ followed by a CRI of 100 µg kg^−1^ minute^−1^;

Group DEX: Dexmedetomidine (Dexdomitor; Orion, Finland) IV LD of 2 µg kg^−1^ followed by a CRI of 2 µg kg^−1^ hour^−1^;

Group LIDO-DEX: Lidocaine IV LD of 2 mg kg^−1^ followed by a CRI of 100 µg kg^−1^ minute^−1^, and dexmedetomidine IV LD of 2 µg kg^−1^ followed by a CRI of 2 µg kg^−1^ hour^−1^.

Loading doses were diluted up to a final volume of 3 mL with sterile water and administered IV over 1 minute. Treatments were diluted up to 60 mL with saline 0.9% and delivered as a CRI accordingly. All CRIs were started immediately after bolus administration using a syringe infusion device (Colleague; Baxter Healthcare, IL, USA).

### Statistical analysis

Statistical analysis was performed using SigmaStat 3.5 (Systat Software Inc., Point Richmond, CA, USA). The Shapiro-Wilk test was used for the assessment of data normality. Data are reported as mean ± standard deviation (SD). A repeated-measures ANOVA was used to the evaluate percentage change in MAC_ISO_, and MAC_ISO_ values before (MAC_BASAL_) and after treatment, time to MAC determination and extubation time. ANOVA was used to compare MAC values between treatments. A *post hoc* Tukey test was used where appropriate. Values were considered statistically different when *p*<0.05.

## Results

The mean ± SD MAC_ISO/BASAL_ of all treatments was 1.44±0.04%. MAC_ISO/T_ values are shown in Table 1. MAC_ISO/BASAL_ values were not significantly different among treatments. At the doses administered, LIDO, DEX and LIDO-DEX significantly decreased MAC_ISO_ by 27.3% (range, 12.5–39.2%), 43.4% (33.3–53.3%) and 60.9% (46.1–78.1%) (*p* = 0.001, *p* = 0.013, *p* = 0.013), respectively, when compared to MAC_ISO/BASAL_. The MAC_ISO_ was significantly lower after LIDO-DEX when compared with LIDO or DEX treatments alone (*p* = 0.001 and *p* = 0.013, respectively). The time to MAC_ISO/BASAL_ determination was 172±24 min, 194±17 min and 181±18 min for LIDO, DEX and LIDO-DEX, respectively; these values were not significantly different between groups. The time for MAC_ISO_ determination after LIDO, DEX and LIDO-DEX were 168±26 min, 152±10 min and 163±15 min, respectively. These values were not significantly different when the groups were compared. Extubation time was 9.0±1 min, 9.7±1 min and 10.6±1 min for groups LIDO, DEX and LIDO-DEX, respectively (*p* = 0.057). Anaesthetic recovery was uneventful in all dogs. Blood gas values were not significantly different between groups for pH, PaCO_2_ or PaO_2_. Other data are reported in Table 2. HR was significantly lower in DEX (*p*<0.001) and LIDO-DEX (*p*<0.001) when compared with baseline. HR was significantly lower in LIDO-DEX when compared with LIDO (*p* = 0.009) and DEX (*p* = 0.002). SAP was significantly higher in LIDO-DEX than under basal conditions (p = 0.04), LIDO (*p* = 0.004) and DEX (*p* = 0.04).

**Table 1:** Mean ± SD of minimum alveolar concentration of isoflurane (MAC_ISO_) before (MAC_ISO/BASAL_) and after one of the following treatments (MAC_ISO/T_): lidocaine (LIDO), dexmedetomidine (DEX), or the combination LIDO-DEX in dogs (*n* = 7).

Treatment	MAC_ISO/BASAL_	MAC_ISO/T_	% Change in MAC_ISO_ (% range)
LIDO	1.28±0.16%.	0.93±0.11*	27.3 (12.5–39.2)*
DEX	1.58±0.28%.	0.90±0.17*	43.4 (33.3–53.3)*
LIDO-DEX	1.46±0.21%.	0.57±0.18* #	60.9 (46.1–78.1)* #

The percentage (%) change in MAC_ISO_ after treatment was calculated from [(MAC_ISO_ after treatment – MAC_ISO/BASAL_)/MAC_ISO/BASAL_] X 100.

*Statistically different from MAC_ISO/BASAL_ (*p*<0.05).

# Statistically different from the rest of the treatments (*p*<0.05).

**Table 2:** Mean ± SD of cardiovascular parameters and other variables measured immediately before the MAC determination of isoflurane (MAC_ISO/BASAL_) and immediately before the final MAC of isoflurane (MAC_ISO/T_) determination during constant rate infusion of lidocaine (LIDO), dexmedetomidine (DEX), or the combination LIDO-DEX.

		Treatment		
Variables	ISO/BASAL	LIDO	DEX	LIDO-DEX
Heart rate (beats min^−1^)	107±4	97±7	74±7	62±7#
Systolic blood pressure (mmHg)	102±18	103±15.8	106±10.4	128±12.6#
Diastolic blood pressure (mmHg)	64±12.4	63±11.3	64±11.3	72±8.3
Mean arterial pressure (mmHg)	78±13.4	77±12.6	79±9.7	93±9.9
Pulse oximetry values (SpO_2_%)	97±0.3	97±0.5	97±0.5	96±0.5
Oesophageal temperature (°C)	37.8±0.1	38±0.1	38.1±0.1	38.3±0.1
pH	7.33±0.028	7.36±0.032	7.31±0.021	7.31±0.021
PaCO_2_ (mmHg)	36.8±2.63	35.0±3.1	35.1±4.24	36.1±3.46
PaO_2_ (mmHg)	503.2±28.19	487.6±36.14	486.5±50	501.33±22.85

# Statistically different to baseline and to the rest of the treatments (*p*<0.05).

## Discussion

The results of this study are consistent with previous reports that showed a decrease in MAC_ISO_ after the administration of lidocaine or dexmedetomidine [3], [4], [21], [22]. In addition, the combination LIDO-DEX produced a greater effect than the two drugs alone, in accordance with the hypothesis of this study. The range of MAC values reported here for isoflurane (1.28–1.58%) was similar to that shown in previous studies (1.34–1.57%) [2], [3], [4], [17]. One reason for small discrepancies among MAC studies is subjectivity in the interpretation regarding what constitutes movement after the application of a noxious stimulus. However, bias was minimised by the use of the same anatomical site and the same noxious stimulation in all dogs. In addition, a single observer was employed to determine gross movement during the MAC determination. Nevertheless, MAC values recorded in this study may have been subject to small errors [23]. LIDO is a sodium channel blocker that reduces inhalant MAC by unknown mechanisms [3]. IV administration of lidocaine has been shown to reduce MAC_SEVO_ in dogs [7], [9] and MAC_ISO_ in several species in a dose-dependent manner. Cardiovascular changes such as slight non-significant increases in blood pressure have been observed in different species, but are considered to be of minimal clinical relevance [2], [3], [8], [24], [25], in agreement with the results obtained in our study. The design of the present study was not able to demonstrate that a significant reduction in MAC_ISO_ after LIDO administration was associated with significant changes in heart rate or blood pressure.

In the present study, a lidocaine CRI of 100 µg kg^−1^ minute^−1^ reduced MAC_ISO_ by approximately 27%, which is similar to the results in previous reports. When LIDO was administered to dogs (LD of 2 mg kg^−1^ followed by a CRI of 50 or 200 µg kg^−1^ minute^−1^), the drug reduced the MAC_ISO_ by 18.7% and 43.4%, respectively [3]. Lidocaine CRI (LD 1.5 mg kg followed by 250 µg kg^−1^ minute^−1^) reduced ISO requirements by 34–44% in dogs undergoing unilateral mastectomy, which may be of clinical relevance [5].

Dexmedetomidine is the most selective α_2_ adrenergic agonist commonly used in the clinical setting due to its sedative and analgesic effects [6], [26], as well as for its ability to reduce the anaesthetic requirements for the induction and maintenance of general anaesthesia [4], [6]. In the present study, at the dose administered, DEX decreased MAC_ISO_ by 43.4%, similar to what was observed in a previous study [4]. DEX reduced MAC_ISO_ by 18% and 59% after an LD of 0.5 µg kg^−1^ followed by CRI at 0.5 µg kg^−1^ hour^−1^, and an LD of 3 µg kg^−1^ followed by CRI at 3 µg kg^−1^ hour^−1^, respectively [4]. DEX increases systemic blood pressure, presumably as a result of α_2_-mediated vasoconstriction [4]. Baroreflex-mediated bradycardia is commonly observed due to increased vagal tone, decreased sympathetic tone and peripheral vasoconstriction [27], [28]. According to our results, DEX alone did not increase the blood pressure as expected; even when MAC_ISO_ after DEX CRI was significantly lower than MAC_ISO/BASAL_, only a slight increase in arterial blood pressure was recorded. However, there was a significant increase in SAP and a decrease in HR with the combination LIDO-DEX when compared with MAC_ISO/BASAL_. This finding may be related to significantly decreased isoflurane concentrations, as reported previously [3], [7]. The dosage regimens reported here are commonly used during surgery in dogs when the provision of analgesia, sedation and limitation of the stress response is required [6], [11], [17].

The combination of LIDO and DEX produced a statistically significant reduction in MAC_ISO_, of 60.9%. Even though a mathematical reduction in MAC_ISO_ of almost 70% could be expected (27% LIDO plus 43% DEX), the design of the current study did not make it possible to determine if an additive or synergistic effect between LIDO and DEX was present. Some pharmacological variables such as chemical drug interactions involving pH and the degree of ionisation, which were not considered in this study, may have influenced the effect of the drug combination. However, it is clear that some combined effect was observed, since the combination of drugs produced MAC_ISO_ reductions that were greater than when LIDO or DEX was administered alone. Indeed, the combination of different analgesic drugs has been shown to reduce inhalant anaesthetic requirements in the clinical setting. Based on the results of the present study, it can be concluded that the combination of LIDO and DEX may be used to substantially decrease ISO requirements in dogs undergoing surgery.

In conclusion, this study showed that, at the doses administered, LIDO and DEX reduced MAC_ISO_ in the same trend as reported by previous studies. The combination of these treatments resulted in a greater MAC_ISO_ reduction than the two drugs alone. Their use, at the doses studied, provides a clinically important reduction in the concentration of ISO during anaesthesia in dogs. Further study is needed to clarify the interaction of LIDO and DEX with ISO and their cardiovascular effects.
